# Morphological performance and seasonal pattern of water relations and gas exchange in *Pistacia lentiscus* plants subjected to salinity and water deficit

**DOI:** 10.3389/fpls.2023.1237332

**Published:** 2023-09-05

**Authors:** Sara Álvarez, Jose Ramon Acosta-Motos, María Jesús Sánchez-Blanco

**Affiliations:** ^1^ Unidad de Cultivos Leñosos y Hortícolas, Instituto Tecnológico Agrario de Castilla y León (ITACyL), Valladolid, Spain; ^2^ Grupo de Biotecnología Vegetal para la Agricultura y la Alimentación (BioVegA), Universidad Católica San Antonio de Murcia, Murcia, Spain; ^3^ Departamento de Riego, Centro de Edafología y Biología Aplicada del Segura (CEBAS-CSIC), Murcia, Spain

**Keywords:** biomass accumulation, gas exchange, ion uptake, irrigation, mineral distribution, osmotic adjustment, principal component analysis, water status

## Abstract

Soil water deficit and salinity represent a major factor impacting plant survival and agricultural production. The frequency and severity of both abiotic stresses are expected to increase in a context of climate change, especially in arid and semi-arid regions. This work studied the growth pattern, biomass and mineral distribution and the seasonal pattern of water status, photosynthetic rate and stomatal conductance in plant of *Pistacia lentiscus* grown under different levels of water deficit and salinity. *P. lentiscus* plants growing under greenhouse conditions were subjected to four irrigation treatments during 11 months: control (C, 1 dS m^-1^), moderate water deficit (MW, 1dS m^-1^, 60% of the control), severe water deficit (SW, 1 dS m^-1^, 40% of the control) and saline (S, 4dS m^-1^). The results show that *Pistacia lentiscus* plants were more affected by deficit irrigation than salinity. Deficit irrigation and salinity inhibited plant height, with reductions of 20%, 22% and 35% for S, MW and SW, respectively. Total leaf area was not modified by effect of the treatments, with the result that plant compactness increased in MW. The salt stressed plants only showed lower relative growth rate at the end of the experiment. Plants responded to saline or drought stress by increasing their osmotic adjustment, which was more pronounced under salinity. Saline plants had the highest values in Na^+^ and Cl^-^ ions and the lowest values for K^+^/Na^+^ and Ca^2+^/Na^+^ ratios in leaves and stems, which is correlated with a decrease in growth, stomatal conductance, photosynthesis and stem water potential, and can be used as a diagnostic tool to assess plant tolerance to salinity stress. As a measure of plant hydration, relative water content was more sensitive to deficit irrigation than salinity, being a good indicator of water stress. *P. lentiscus* plants subjected to both deficit irrigation treatments exhibited an increase in their intrinsic water use efficiency, which is an important adaptation for plants growing in environments with water scarcity.

## 1 Introduction

The escalation of both the frequency and magnitude of water stress and salinity is foreseen to manifest in numerous global regions within the framework of ongoing climate change (Chaudhry and Sidhu, 2022; Soares et al., 2022). Rising temperatures and changing precipitation patterns are projected to exacerbate existing water scarcity issues, while sea level rise and changing groundwater dynamics are expected to increase the incidence of salinity intrusion in coastal areas (Asif et al., 2023). As water stress and salinity stress become more common the impacts on agriculture, ecosystems, and human well-being are likely to become more severe (Semeraro et al., 2023). In response to these challenges, there is a growing need to develop adaptive strategies to mitigate the impacts of water and salinity stresses. These strategies may include improving water use efficiency, developing crop varieties that are more resistant to drought and salinity, encouraging sustainable land management strategies, and allocating resources towards the development of water treatment and desalination technologies are essential steps in fostering sustainable water resource utilization (Acosta-Motos et al., 2020; Albatayneh, 2023; Patel et al., 2023).

Water stress occurs when plants experience a lack of water, either due to insufficient rainfall, high temperatures, or other factors that increase evaporation. This can lead to reduced plant growth, leaf wilting, and even death in severe cases. Plants respond to water stress by reducing their stomatal openings to conserve water, which can also affect the photosynthetic process and reduce their ability to grow (Arve et al., 2011; Bodner et al., 2015). Salinity stress occurs when plants are exposed to high levels of salt in the soil or water. High salinity can lead to reduced water uptake by plants, which in turn can lead to reduced plant growth, wilting, and even death. Plants respond to salinity stress by developing mechanisms to exclude or tolerate salt, such as increased root growth or salt secretion (Acosta-Motos et al., 2017; Acosta-Motos et al., 2020). Both water stress and salinity can have significant impacts on agricultural productivity and ecosystem health. In some cases, plant breeders have developed crop varieties that are more resistant to these stresses, but in other cases, it may be necessary to implement management practices such as irrigation scheduling, soil amendments, or alternative crops to mitigate their effects (Paranychianakis and Chartzoulakis, 2005; Phogat et al., 2020).

*Pistacia lentiscus*, also known as mastic tree or lentisk, has potential uses in a context of climate change. As a drought-tolerant species, it is likely to be more resilient to water stress than many other ornamental plants, making it a good choice for landscaping in regions prone to drought (Bussotti et al., 2014; Leotta et al., 2023). Also, its deep roots and ability to thrive in poor soils make it a potentially useful species for erosion control and land restoration projects (Ramón Vallejo et al., 2012). In addition to its potential uses in a context of water stress and drought, *Pistacia lentiscus* may also have applications in regions affected by salinity stress. The species is known for its ability to tolerate high levels of salt in the soil and can even grow in saline soils near the coast. In areas affected by salinity stress, the use of *Pistacia lentiscus* as an ornamental plant in landscaping projects may be particularly valuable (Álvarez et al., 2018; Castillo-Campohermoso et al., 2020). The plant's tolerance to salt and its ability to thrive in poor soils make it a good choice for revegetation and erosion control projects in coastal areas (Parraga-Aguado et al., 2013). By incorporating *Pistacia lentiscus* into agroforestry systems, farmers may be able to improve soil structure, reduce soil salinity, and improve water use efficiency, leading to higher crop yields and greater resilience in the face of climate change. Finally, the species has been successfully integrated into agroforestry systems in Mediterranean regions, where it has been used for shade, windbreaks, and as a source of animal fodder (Williams, 2002). It is well-known that analysing individual stresses, such as water supply restrictions and salt stress, rather than their combination, can provide several advantages when studying their effects on plants. Studying individual stresses allows to isolate and understand the specific effects of each stress factor. This can help in determining the unique physiological and morphological responses of *Pistacia lentiscus* plants to each stressor drought stress and salinity. The purpose of this work was to study growth pattern, biomass and mineral distribution and the seasonal pattern of water status, photosynthetic rate and stomatal conductance in plant of *Pistacia lentiscus* grown under deficit irrigation and salinityto determine the ability and response of plant to cope with water and saline stresses, which represents a major factor in plant growth and survival in a context of climate change.

## 2 Materials and methods

### 2.1 Plant and experimental conditions

*Pistacia lentiscus* L. plants (180) were grown in 15 x 15 x 20 cm pots (4L) filled with a substrate blend comprising coconut fibre, black peat mixed with sphagnum peat, and perlite of 5:4:1, enriched with Osmocote plus fertilizer (2 g L^-1^ substrate) containing a balanced formulation of nitrogen (N), phosphorus (P), potassium (K), and microelements (14:13:13N, P, K plus microelements). The experimental investigation took place within a controlled greenhouse environment, which was previously detailed in the methodology section in Álvarez et al. (2018). Temperature values (T^a^) ranged between 2.5 and 37.9 °C and relative humidity (RH) values oscillated between 20.3 and 90.7%. A mean temperature of 20.3°C and relative humidity (RH) of 62.9% was registered during the experimental period. Before commencing the treatments, all plants were consistently irrigated to reach field capacity on a daily basis for a period of four weeks.

### 2.2 Water irrigation treatments

After acclimating the *P. lentiscus* plants to greenhouse conditions, (a total of 45 plants per treatment) were subjected to an experimental duration of up to 11 months, from December 2007 to November 2008. Four distinct irrigation treatments were implemented, including a control treatment where plants received watering up to 100% of the water holding capacity, with 15% leaching (v/v) using tap water of electrical conductivity (EC) measuring 1.0 dS m^-1^. Additionally, a saline water treatment was employed, maintaining the water holding capacity at 100% while incorporating salt to attain a concentration of 44 mM Na Cl (4.0 dS m^-1^). Furthermore, two deficit irrigation treatments were implemented, known as moderate deficit irrigation (MW) and severe deficit irrigation (SW). These treatments involved the application of 60% and 40% of the water quantity supplied in the control treatment, respectively. All the plants were watered subjected to daily watering throughout the experimental period.

### 2.3 Leaf mineral content

The inorganic solute concentrations were measured at the end of the experimental period in the same plants used for the biomass parameters calculation. The concentrations of Ca^2+^, K^+^ and Na^+^, Mg^2+^, P, S, Mn, B and Zn ions were measured using a digestion extract of 100 mg of tissue powder with 50 ml of a mix of HNO_3_:HCl O_4_ (2:1, v/v) by using an inductively coupled plasma optical emission spectrometer (ICP-OES-IRIS intrepid II XDL, Thermo Fisher Scientific Inc., Lough-borough, UK). The concentration of Cl^-^ ions was performed utilizing a chloride analyser (Model 926, Sherwood Scientific Ltd., Cambridge, UK).

### 2.4 Plant growth, ornamental traits

Upon reaching the conclusion of the experimental period, the substrate surrounding the roots of ten plants per treatment was meticulously rinsed away. The plants were then carefully separated into their constituent parts, including leaves, stem and roots. Subsequently, they were subjected to oven-drying at a temperature of 80°C until an invariable weight was achieved, enabling the measurement of their respective dry weights (DW). Leaf area was assessed in the identical set of plants, by employing a leaf area meter (Delta-T Devices Ltd., Cambridge, UK). Specific leaf area (SLA) was computed by dividing the leaf area by the corresponding leaf dry weight, while leaf area ratio (LAR) was determined by dividing the leaf area by the total dry weight. Plant height was periodically measured in a sample of 25 plants per treatment and was taken as the vertical distance from substrate to the highest leaf. Relative growth rate (RGR) was calculated by determining the rate of height increase per unit of initial plant height. Compactness, on the other hand, was calculated dividing the leaf area by the corresponding plant heights. Leaf colour measurements were conducted using a Minolta CR-10 colorimeter, which facilitated the assessment of colour coordinates such as lightness (L*), chroma (C*) and hue angle (h°) following the methodology established by McGuire (1992). A total of seven plants per treatment were utilized for this analysis.

### 2.5 Water status

During the course of the experiment, various physiological parameters related to leaf water status were assessed. These parameters included leaf water potential (Ψ_l_) at predawn and midday, leaf turgor potential (Ψ_t_), leaf osmotic potential (Ψ_o_), leaf osmotic potential at maximum saturation (Ψ_100s_), stem water potential (Ψ_s_) at midday and relative water content (RWC) at predawn and midday. Eight plants per treatment were selected, and measurements were performed on mature leaves at midday. The estimation of Ψ_l_ was carried out using the method outlined by Scholander et al. (1965), employing a pressure chamber (Soil Moisture Equipment Co, Santa Barbara, CA, USA). Leaves were promptly placed in the chamber within 20 seconds of collection and pressurised at a rate of 0.02 MPa s^−1^ (Turner, 1988). The Ψ_t_ was calculated as the difference between leaf water and leaf osmotic potential. Osmotic potential was determined using a Wescor 5520 vapor pressure osmometer (Wescor Inc., Logan, UT, USA). Freshly cut leaves were wrapped in aluminum foil, subjected to cell membrane rupture by liquid nitrogen, and stored at −30 °C. Thawed leaves were squeezed to extract a drop for osmotic potential measurement. To determine the osmotic potential at maximum saturation (Ψ_100s_), the leaves were immersed in distilled water at 4°C in the dark for 24 hours until they reached maximum turgor. Following the removal of excess water with filter paper, the leaves were wrapped in aluminium foil and frozen in liquid nitrogen as described earlier for osmotic potential measurement (Gucci et al., 1991). Ψ_s_ was measured in leaves that had been covered with both a plastic sheet and aluminium foil for at least 2 hours before measurement to minimize leaf transpiration. This ensured that leaf water potential equalled stem water potential (Begg and Turner, 1970). The RWC of leaves was calculated following the method described by Barrs (1968).

### 2.6 Gas exchange and photosynthesis parameters

During the course of experiment, the fluctuations in leaf stomatal conductance (g_s_) and net photosynthetic rate (P_n_) were evaluated in mature leaves at midday. Measurements were performed on eight plants per treatment using a gas exchange system (LI-6400, LI-COR Inc., Lincoln, NE, USA). Leaf gas exchange was measured on young, fully expanded leaves, placed in a 2 cm^2^ leaf cuvette. The CO_2_ concentration in the cuvette was maintained at 400 µmol mol^-1^ (≈ ambient CO^2^ concentration). Measurements were performed at a saturating light intensity subindice of 1200 µmol m^-2^ s^-1^ and at ambient temperature and relative humidity. The g_s_ reflects the rate at which stomata open and close, allowing the exchange of gases between the leaf and the atmosphere, while P_n_ represents the rate of carbon dioxide uptake during photosynthesis. Additionally, the P_n_/g_s_ ratio, calculated as the ratio of net photosynthetic rate to stomatal conductance, was utilized as an indicator of the intrinsic water use efficiency, providing insights into the plant's ability to balance water loss and carbon assimilation.

### 2.7 Statistics

The plants were arranged in a randomized block design and placed on crop benches. Each of the four treatments, namely control (C), moderate irrigation treatment (MW), severe irrigation treatment (SW) and salt treatment (S) was divided into three blocks. Within each block, 15 plants were randomly allocated resulting in a total of 45 plants per treatment. Normality and homoscedasticity of variances were assessed for all variables through the Shapiro–Wilk and Bartlett tests, respectively. To compare the treatments, a one-way analysis of variance (ANOVA) was conducted, followed by a Tukey HSD *post hoc* test. Statistical significance was considered at a threshold of P≤0.05. Stars and rays graphs were generated where each axis represents a specific variable, and the intersection with a vertex of the polygon indicates the relative magnitude of that variable. Additionally, a principal component analysis (PCA) was performed to examine leaf nutrient data. PCA is a statistical method proposed by Pearson (1901) and Hotelling (1933), which aims to describe the variation observed in p random variables using a set of new variables called principal components. These components are uncorrelated with each other and obtained in order of importance. PC1 captures the maximum variation explained by the original variables, while PC2 explains the remaining unexplained variation while being uncorrelated with the PC1, and so on. The selection of principal components was based on eigenvalues greater than or equal to 1.0 (**Supplemental Table 1** and **Supplemental Figure 1**). The statistical analyses were performed using StatGraphics Centurion XV software (StatPoint Technologies, Warrenton, VA, USA).

## 3 Results

### 3.1 Plant quality and mineral distribution throughout the plant

**Figure 1:**
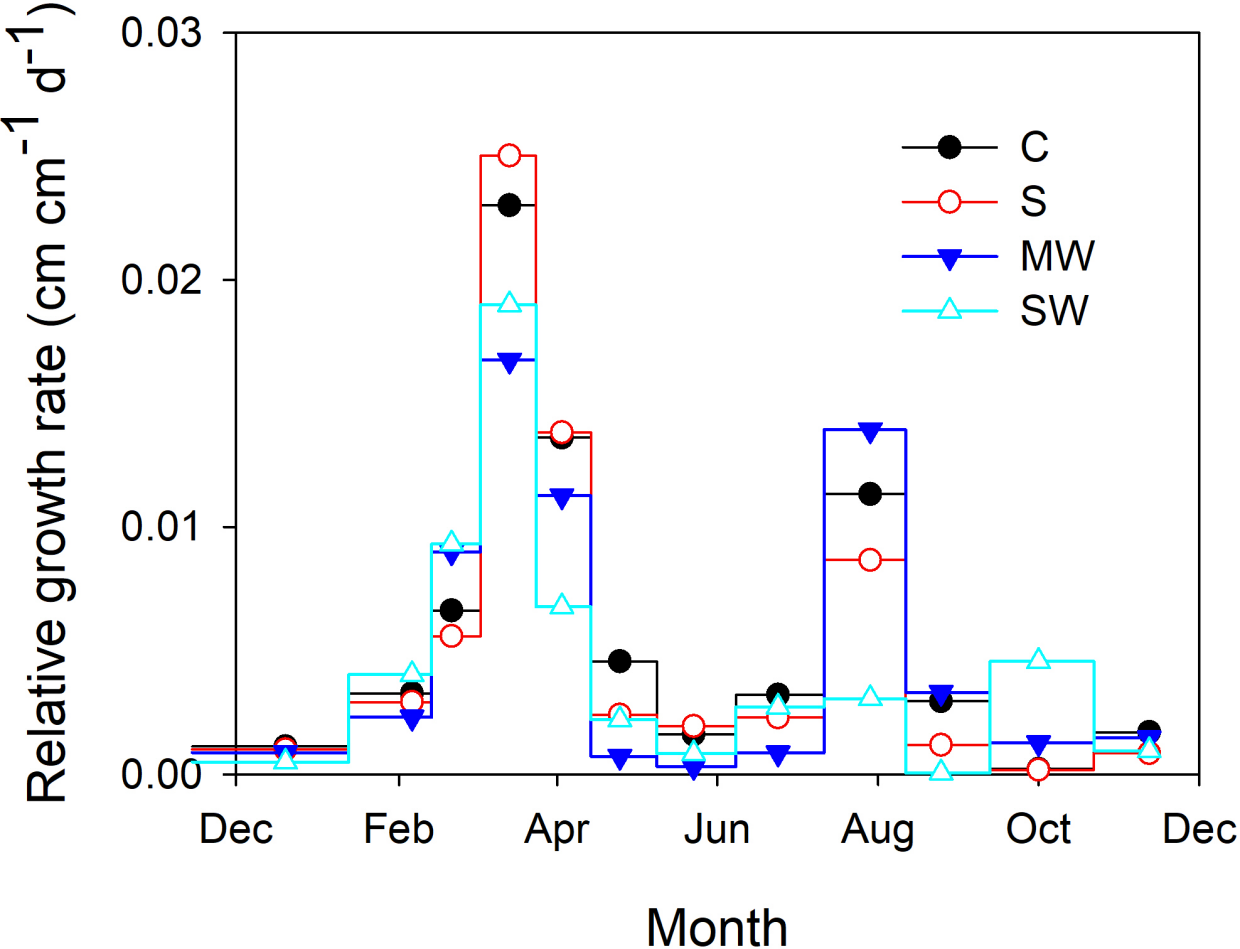
Relative growth rate of *Pistacia lentiscus* plants subjected to saline and deficit irrigation treatments. Values are means of 25 plants per treatment. Symbols represent the different treatments: Control (filled circles), S (open circles), MW (filled triangles) and SW (open triangles).

Upon completion of the experimental duration, no statistically significant disparities were observed in the dry matter accumulation of the *P. lentiscus* plants submitted to saline irrigation (S) and to moderate deficit irrigation (MW) (**Table 1**). However, the severe water deficit treatment (SW) significantly reduced total dry weight (DW) compared the rest of the treatments, while the number of leaves and leaf blade area were not affected by the water-deficit and salinity conditions of the substrate. Total DW of SW-plants was 67% of the control values. The biomass distribution was affected by the water-availability, increased Leaf/Total DW and decreased Stem/Total DW (**Table 1**). Both water deficit treatments significantly increased the leaf area ratio (LAR) compared with control, the effect being more pronounced in SW plants, while the specific leaf area (SLA) was increased in S and SW plants (**Table 1**). Plant height was significantly reduced by salinity and by both water deficit treatments, with reductions of 20%, 22% and 35% for S, MW and SW, respectively; however, total leaf area was not modified, with the result that plant compactness increased in MW (**Table 1**). As regards the evolution of relative growth rate, the relationship between relative growth rate (RGR) and plant height revealed the presence of two distinct growth periods throughout the growing season, specifically in the months of March-April and July-August. This pattern was observed across all plants, despite any other factors that may have influenced plant growth, although both water deficit treatments showed lower RGR in the first growth period and SW plants also in the second growth period, and especially in September (**Figure 1**). The salt stressed plants only showed a lower RGR than the control after the second growth period, at the end of the experiment.

**Table 1:** Growth parameters at the end of the experiment in *P. lentiscus* subjected to different irrigation treatments.

Parameters	Treatments				P
	C	S	MW	SW	
Total DW (g plant^-1^)	61.80±2.92b	62.35±7.12b	53.46±2.27b	41.18±1.53a	**
Leaf blade area (cm^2^)	14.62±1.43	13.62±1.56	12.63±0.79	12.10±1.39	ns
Number of leaves per plant	78.70±7.29	92.00±15.63	103.70±8.84	88.86±6.66	ns
Leaf/total DW (g g^-1^)	26.51±1.42a	26.12±1.58a	32.72±1.36b	32.27±1.31b	**
Stem/total DW (g g^-1^)	0.38±2.17b	0.40±1.36b	0.31±1.84a	0.30±1.31a	***
Root/total DW (g g^-1^)	0.36±2.14	0.33±1.15	0.37±2.13	0.39±1.99	ns
SLA (cm^-2^ g^-1^)	49.80±3.84a	63.87±4.51b	58.92±2.76ab	76.59±3.82c	***
LAR (cm^-2^ g^-1^)	13.20±1.35a	16.88±1.11ab	18.63±1.33b	23.89±1.63c	***
Plant height (cm)	68.95±3.59c	55.40±3.12b	54.15±3.39b	45.40±1.83a	***
Compactness (cm^2^ cm^-1^) cm-1)	16.57±1.47a	22.12±3.36ab	26.06±1.53b	22.70±2.27ab	*

Values are the mean of ten plants, except in plant height, when values are the mean of 25 plants.

Means within a row without a common letter are significantly different according to Duncan 0.05 test.

(P; probability level, ns; non significance, *P<0.05, ** P ≤0.01, *** P ≤ 0.001).

**Figure 2:**
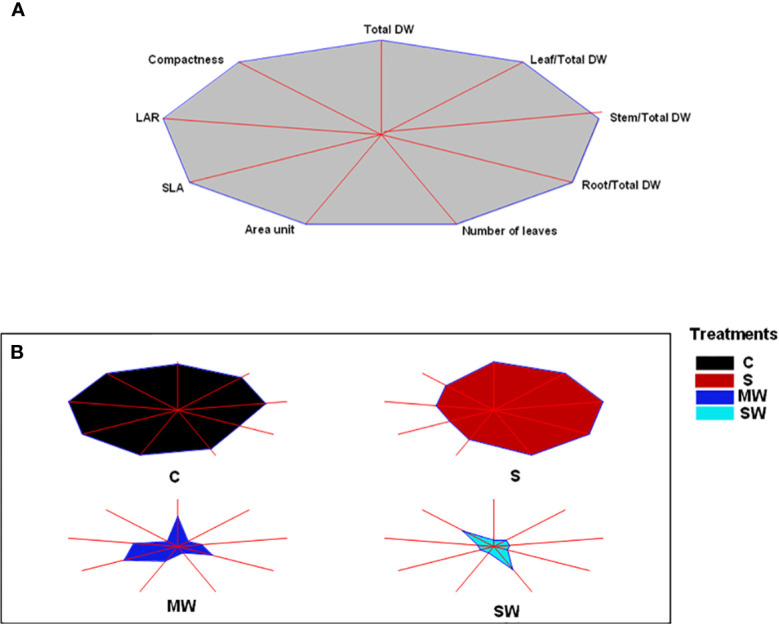
Stars and rays graphs displaying the differences between accessions for the morphological traits analyzed. Each axis represents one variable, and its intersection with a vertex of the polygon indicates the relative magnitude for that variable. **(A)** Reference polygon for variable identification. Leaf area ratio (LAR) and specific leaf area (SLA) are shown as their inverses to visually correlate higher magnitude with a positive trait. **(B)** Individual graphs for each accession. DM, dry matter (%).

To offer a more concise understanding of the effectiveness of each examined treatment, a diagram called a stars and rays graph was created utilizing the key morphological characteristics. To summarize, the greater the distance between the point where each trait's axis intersects with the outer boundary of the diagram and the center of the diagram itself, the greater the magnitude of that trait. This method aids in visually highlighting the variations between the different treatments. For example, Control treatment (C) followed by saline treatment (S) are seen as those treatments that best fulfil the morphological traits studied. On the other hand, the treatments MW and especially SW show small magnitudes for all of the parameters studied, which point the greater capacity of these treatments to respond with morphological changes to the stress imposed (**Figure 2**).

**Figure 3:**
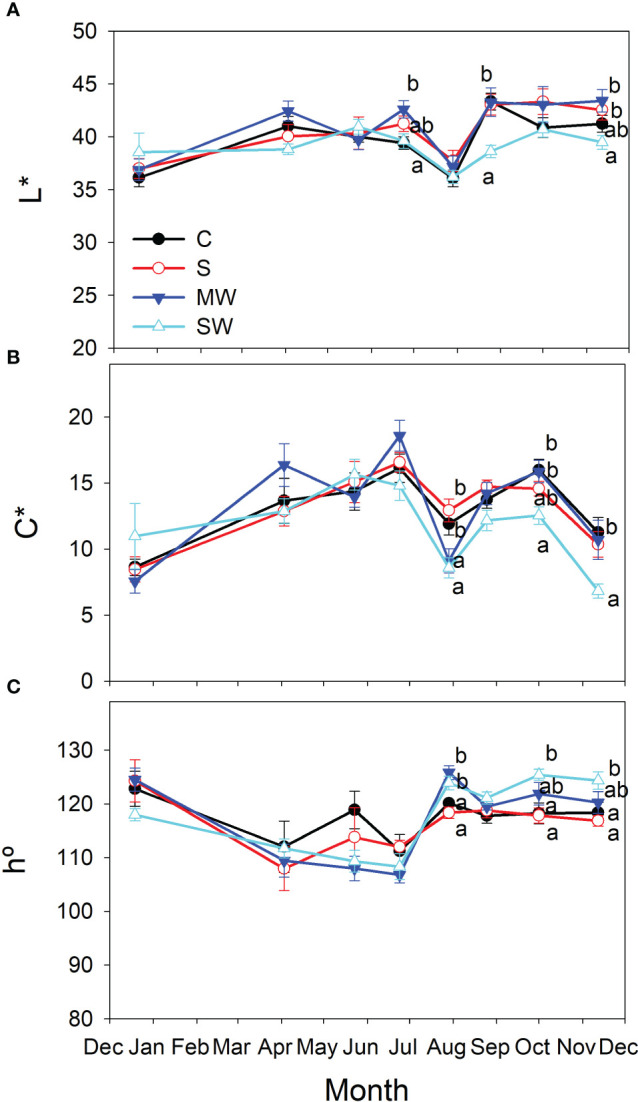
Evolution of leaf color parameters, Lightness [L*; **(A)**], Chroma [C*; **(B)**] and hue angle [h°, **(C)**] of *Pistacia lentiscus* plants subjected to saline and deficit irrigation treatments. Values are means of seven plants per treatment and vertical bars indicate SE. Symbols represent the different treatments: Control (filled circles), S (open circles), MW (filled triangles) and SW (open triangles). Different letters indicate significant differences between treatments according to Duncan t-test (P ≤ 0.05).

There were no discernible alterations in leaf color for MW and S plants when compared to the control group, as indicated in **Figure 3**. However, in plants subjected to SW, higher h° values and lower L* and C* values were recorded at the conclusion of the experiment, confirming a darker and less saturated green hue of the foliage compared to the control plants (**Figure 3**).

**Figure 4:**
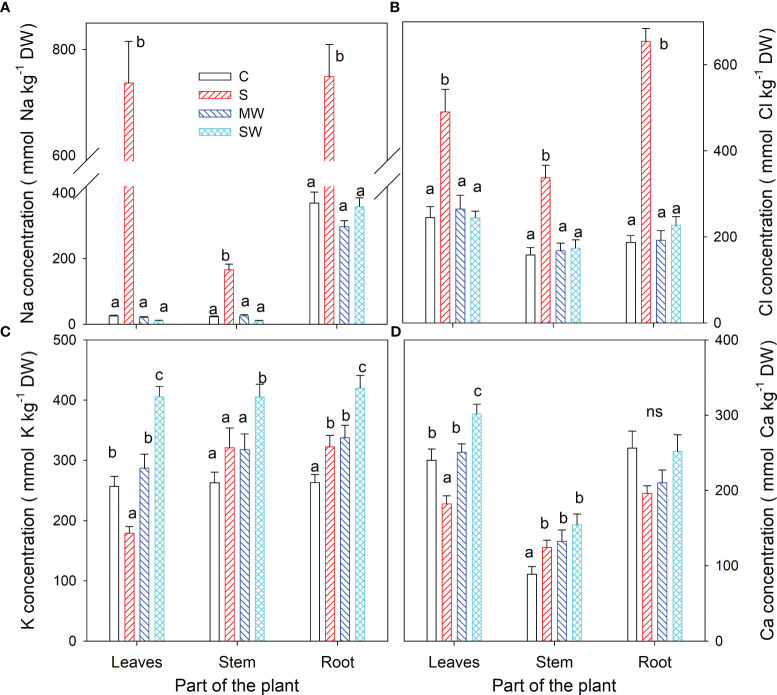
Concentrations of Na^+^ **(A)**, Cl^-^ **(B)**, K^+^ **(C)** and Ca^2+^ **(D)** at the end of the experiment in *Pistacia lentiscus* plants subjected to saline and deficit irrigation treatments. Values are means of ten plants per treatment and vertical bars indicate SE. Different letters within a part of the plant indicate significant differences between treatments according to Duncan t-test (P ≤ 0.05).

**Figure 4** displays the concentrations of Na^+^, Cl^-^, K^+^, and Ca^2+^ in the leaves, stems, and roots at the conclusion of the experimental period. Notably, no noticeable buildup of Na and Cl was detected in plants exposed to either water stress treatment. However, the concentration of both ions exhibited an upward trend in all plant components as salinity levels increased, as depicted in **Figures 4A, B** The K^+^ concentration decreased to 70% in the leaves of S plants compared to control plants, whereas an increase was observed in the root of these plants (up to 22%). The K^+^ concentration increased with severe water deficit in all parts of the plants (up to 55-60%) (**Figure 4C**). With respect to Ca^2+,^ its concentration decreased in the leaves of S plants and increased by 40, 49 and 74% in stem of S, MW and SW plants (**Figure 4D**). Next, in all plant components, the ratios of K^+^/Na^+^ and Ca^2+^/Na^+^ were observed to decline due to saline treatment, while in severe water deficit treatment these ratios were higher than in control plants (**Table 2**). Correlation between different growth parameters (total DW, plant height, SLA and compactness) and Na^+^ and Cl^-^ are shown in **Table 3**, the plant height reduction was significantly related with Na^+^ and Cl^-^ concentration. The augmentation of specific leaf area (SLA) exhibited a strong positive correlation with both Na^+^ and Cl^-^ concentrations, with a higher correlation coefficient (r) observed for Cl^-^. No significant correlations were observed between any other parameters examined in the study (**Table 3**).

**Table 2:** Leaf, stem and root K^+^/Na^+^ and Ca^2+^/Na^+^ ratios at the end of experimental period in *P. lentiscus* subjected to different irrigation treatments.

Parameters		Treatments				P
		C	S	MW	SW	
K^+^/Na^+^	Leaves	10.8±0.9bB	0.3±0.1aA	17.1±3.1bB	42.9±3.0cB	***
	Stem	13.2±1.8bB	2.1±0.4aB	14.8±1.8bB	44.1±3.9cB	***
	Root	0.8±0.1bA	0.5±0.1aA	1.2±0.1cA	1.2±0.1cA	***
		***	***	***	***	
Ca^2+^/Na^+^	Leaves	10.4±1.3bC	0.3±0.1 aA	14.2±1.9bC	32.2±2.6cC	***
	Stem	4.3±0.5bB	0.8±0.1aB	6.4±0.9bB	16.4±1.6cB	***
	Root	0.7±0.1bA	0.3±0.0aA	0.7±0.0bA	0.7±0.0bA	***
		***	***	***	***	

Values are the mean of ten plants.

Means within a row without a common lower case letter are significantly different according to Duncan 0.05 test. Means within a column without a common capital letter are significantly different according to Duncan 0.05 test.

(P; probability level, *** P ≤ 0.001).

**Table 3:** Correlation coefficients (r) between some growth parameters and Na+ and Cl_ concentration in the leaves.

Parameter	Na		Cl	
	r	P	r	P
Total dry weight	-0.122	0.60 ns	-0.014	0.9 ns
Plant height	-0.72	0.003 **	-0.595	0.005 **
SLA	0.64	0.002 **	0.71	0.0004 ***
Compactness	0.11	0.63 ns	0.107	0.65 ns

ns, non significance, **P<0.01, *** P ≤ 0.001.

**Figure 5:**
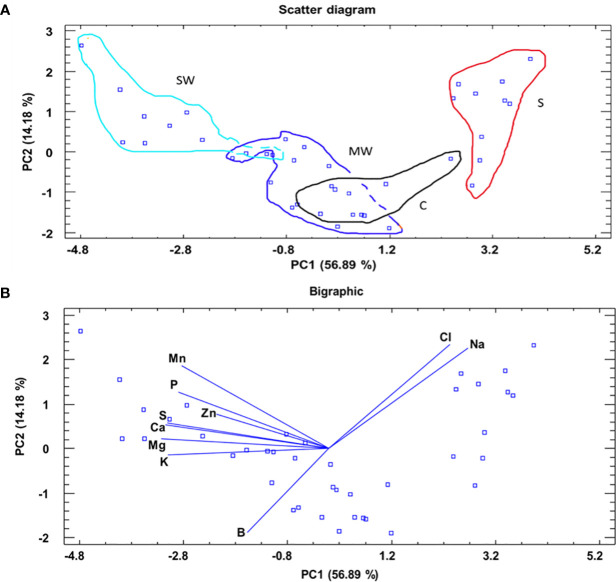
A principal component analysis applied to the different treatments (C, MW, SW and S). A scatter diagram and two principal components (PC1 and PC2) resulted in a model that explained 71.07% of the total variance **(A)**. Bigraphic representation of the principal components marking with lines for each variable and wit points for each score **(B)**.

Furthermore, to assess the distinct separation of the various treatments based on leaf nutrient levels at the conclusion of the experiment, a principal component analysis (PCA) was performed. The scatter diagram (**Figure 5A**) and bigraphic (**Figure 5B**) figures were obtained by the principal component table. In addition, the average score for each of the four treatments was added (**Supplemental Table 2A**). The analysis in the scatter plot and biographic revealed a notable detach of the treatments using the two principal components (PCs). However, PC1, which accounts for 56.89% of the experimental variability, played a particularly significant role in the observed separation, was better, with a value of F = 89.69***, classifying the treatments into four clusters good separated from left to right (SW, MW, C and S) (**Supplemental Tables 2B, C**). Additionally, PC2, which accounts for 14.18% of the experimental variability, also demonstrated a substantial ability to separate the treatments. This was further supported by a statistically significant F value of 17.38***, classifying the treatments in two clusters (S and SW) and (MW and C) (**Supplemental Tables 2D, E**). For PC1, the variables or nutrients most important with more weight (variables with a higher absolute value) were: Mg^2+^ and Ca^2+^. For PC2, the variables or nutrients most important with more weight were: Cl^-^, Na^+^ and B (**Supplemental Table 3**).

### 3.2 Physiological measurements: seasonal pattern of water status and gas exchange

**Figure 6:**
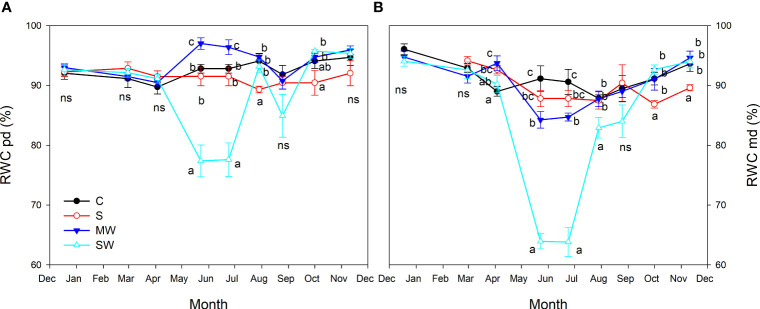
Evolution of leaf relative water content (RWC) at predawn **(A)** and midday **(B)** of *Pistacia lentiscus* plants subjected to saline and deficit irrigation treatments. Values are means of eight plants per treatment and vertical bars indicate SE. Symbols represent the different treatments: Control (filled circles), S (open circles), MW (filled triangles) and SW (open triangles). Different letters indicate significant differences between treatments according to Duncan t-test (P ≤ 0.05). ns, non significance.

As regards the seasonal pattern of leaf relative water content (RWC), for the majority of the experiment (first seven months), no significant differences were observed between the control plants and those subjected to saline treatment, as depicted in **Figures 6A, B**. However, towards the end of the experiment, the impact of salinity became evident as indicated by reduced values of relative water content, especially at midday. In both deficit irrigation plants RWC at midday was reduced in summer (**Figure 6B**), but was much more marked under SW, reaching minimum values in May and June (64%). After this period (as of July) RWC midday showed a recovery in both water deficit plants and they reached the control values at the end of the experiment. However, at predawn, both levels of deficit irrigation had opposite effects on RWC (May and June). SW led to a decrease in RWC in the early summer and moderate water deficit led to an increase in RWC compared with control (**Figure 6A**).

**Figure 7:**
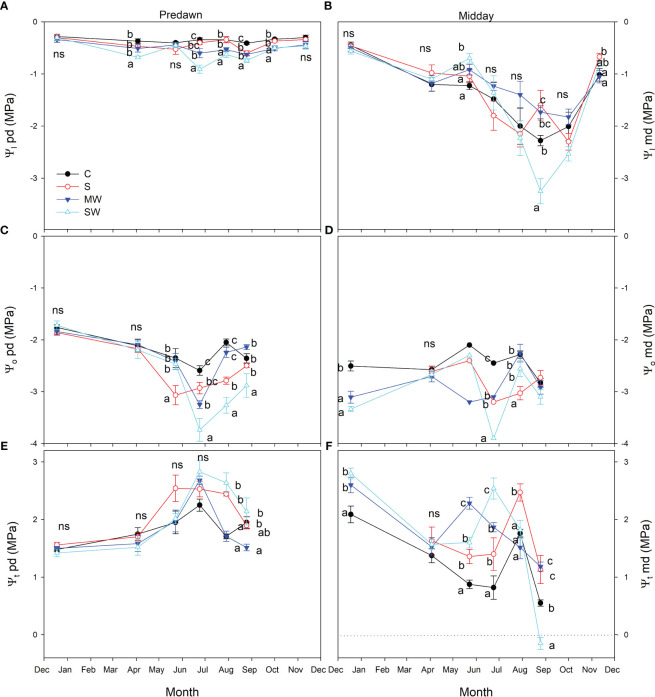
Evolution of the leaf water potential at predawn [Ψ_l_ pd, **(A)**], leaf osmotic potential at predawn [Ψ_o_ pd; **(B)**], leaf turgor potential at predawn [Ψ_t_ pd; **(C)**], leaf water potential at midday [Ψ_l_ md, **(D)**], leaf osmotic potential at midday [Ψ_o_ md, **(E)**] and leaf turgor potential at midday [Ψ_t_ md, **(F)**] of *Pistacia lentiscus* plants subjected to saline and deficit irrigation treatments. Values are means of eight plants per treatment and vertical bars indicate SE. Symbols represent the different treatments: Control (filled circles), S (open circles), MW (filled triangles) and SW (open triangles). Different letters indicate significant differences between treatments according to Duncan t-test (P ≤ 0.05). ns, non significance.

Additionally, this trend is evident in the seasonal variations of predawn leaf water potential (Ψ_l_) values, as depicted in **Figure 7A**. Overall, the control plants exhibited higher leaf water potential values compared to the stressed treatments, indicating a noticeable difference in water status between the two groups, the SW treatment showing the lowest values in June, around -0.9 MPa. Throughout the course of the experiment, the plants subjected to the saline treatment displayed intermediate Ψ_l_ values at predawn between control and MW, suggesting that saline treatment induced a reduced level of osmotic stress than MW. The presence of salinity and deficit irrigation resulted in a decrease in leaf osmotic potential at predawn, with a more pronounced effect observed in the SW treatment, as depicted in **Figure 7B**. This, in turn, led to higher values of leaf pressure potential specifically in the SW treatment (**Figure 7C**) and occasionally in S plants. Leaf water potential at midday (Ψ_l_) exhibited a declining trend across all treatments in response to increasing atmospheric evaporative demand. The highest Ψ_l_ values were recorded in December, while the lowest values occurred in August. Notably, the SW treatment demonstrated the most extreme reduction in Ψ_l_, reaching values as low as approximately -3.25 MPa during August (**Figure 7D**).

The differences between treatments in Ψ_l_ midday values were consistently lower than the predawn values, primarily because of the influence of environmental factors (**Figure 7D**). Throughout the experimental period, no significant variations in Ψ_l_ levels were observed among the treatments, even though the substrate moisture was clearly different, as was reflected in the Ψ_l_ values at predawn. Only in August, plants of severe water deficit treatment showed Ψ_l_ values significantly lower than control (**Figure 7D**). Plants of the MW treatment showed Ψ_l_ values at predawn lower than control during most of the experiment, but these plants showed similar or even higher values than control at midday. Leaf osmotic potential at midday (Ψ_o_) was decreased by salinity and by deficit irrigation (**Figure 7E**), which caused higher values of leaf pressure potential (Ψ_t_) in saline and water stress treatments at midday, except in August, when it resulted in turgor loss in SW plants (**Figure 7F**).

**Figure 8:**
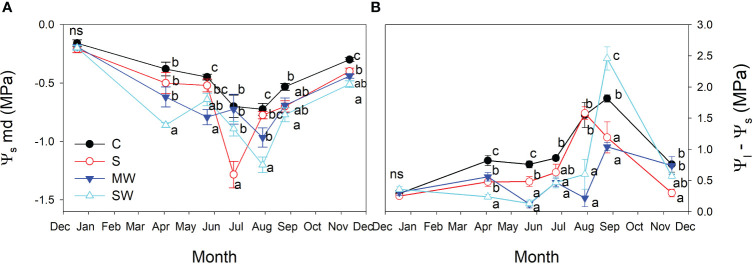
Evolution of the stem water potential [Ψ_s_; **(A)**], and difference between stem and leaf water potential [Ψ_s_-Ψ_l_, **(B)**] of *Pistacia lentiscus* plants subjected to saline and deficit irrigation treatments. Values are means of eight plants per treatment and vertical bars indicate SE. Symbols represent the different treatments: Control (filled circles), S (open circles), MW (filled triangles) and SW (open triangles). Different letters indicate significant differences between treatments according to Duncan t-test (P ≤ 0.05).

**Figure 9:**
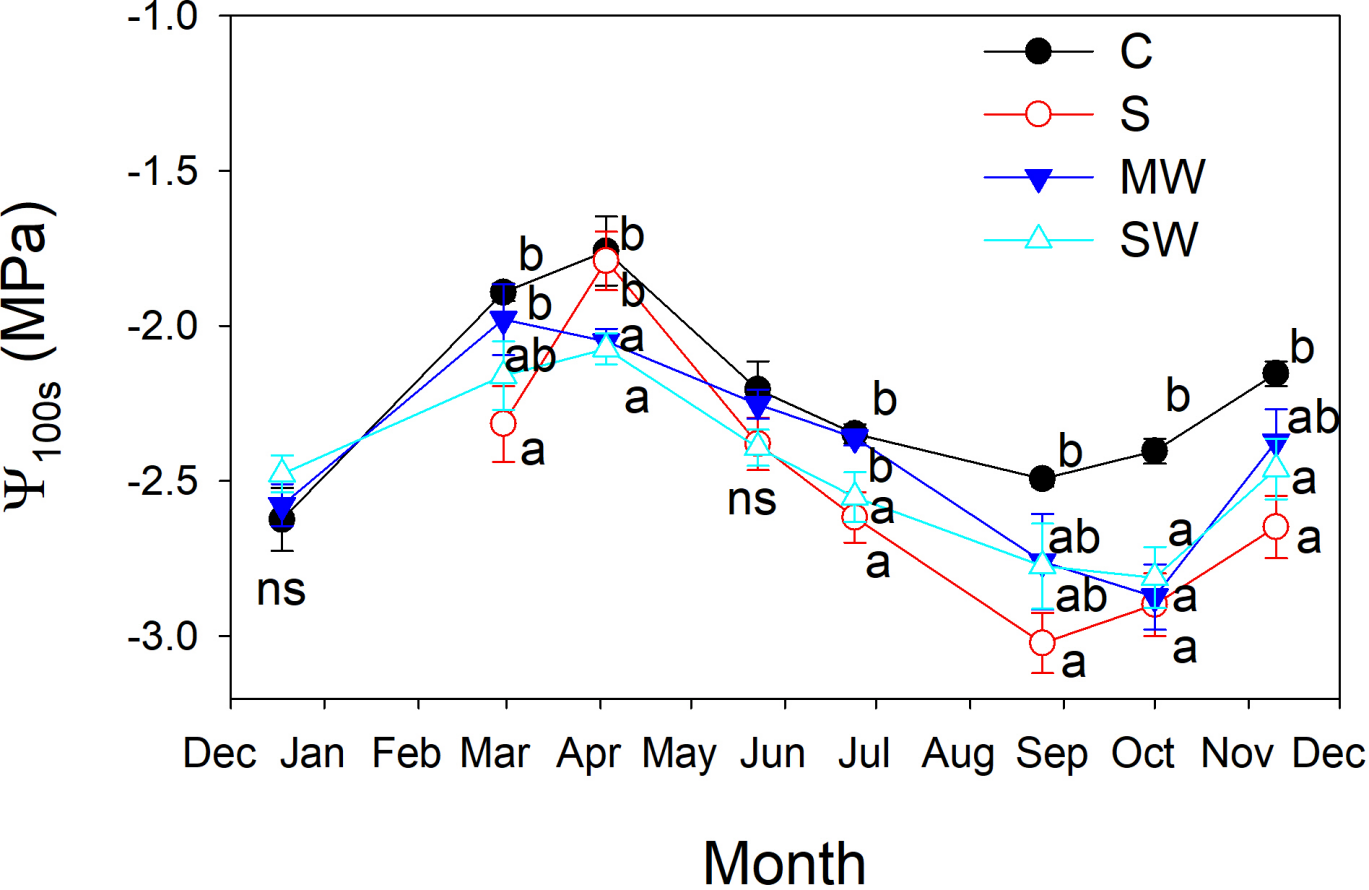
Evolution of the leaf osmotic potential at full turgor (Ψ_100s_) of *Pistacia lentiscus* plants subjected to saline and deficit irrigation treatments. Values are means of eight plants per treatment and vertical bars indicate SE. Symbols represent the different treatments: Control (filled circles), S (open circles), MW (filled triangles) and SW (open triangles). Different letters indicate significant differences between treatments according to Duncan t-test (P ≤ 0.05).

Stem water potential (Ψ_s_) values were notably higher in the control plants compared to the other treatments, and Ψ_s_ values decreased by salinity and water deficit (**Figure 8A**). The standard error associated with stem water potential (Ψ_s_) measurements was considerably lower compared to that of leaf water potential (Ψ_l_). Moreover, significant differences in Ψ_s_ levels were consistently observed between treatments throughout the entire experimental period. These findings indicate that Ψ_s_ served as a more sensitive parameter in identifying significant variations between the treatments than Ψ_l_ measured at midday. Furthermore, it was observed that significant differences between treatments were identified at earlier stages when considering Ψ_s_ compared to Ψ_l_ at predawn, in both salinity and water deficit (**Figure 8**). This indicates again that Ψ_s_ measurements were more effective in detecting treatment distinctions at an earlier time point during the experimental period. The maximum and minimum disparities observe between Ψ_s_ and Ψ_l_ measurements taken simultaneously from the same plant were found to correspond with the maximum and minimum values of g_s_, respectively (**Figure 8B**). In general, the plants of saline and both water deficit treatments showed lower Ψ_100s_ values than control plants throughout the experimental period (**Figure 9**), The variation observed between the values obtained from the control plants and the stressed plants was considered as an estimate of the degree of adjustment (0.22, 0.31 and 0.5 MPa for MW, SW and S, respectively).

**Figure 10:**
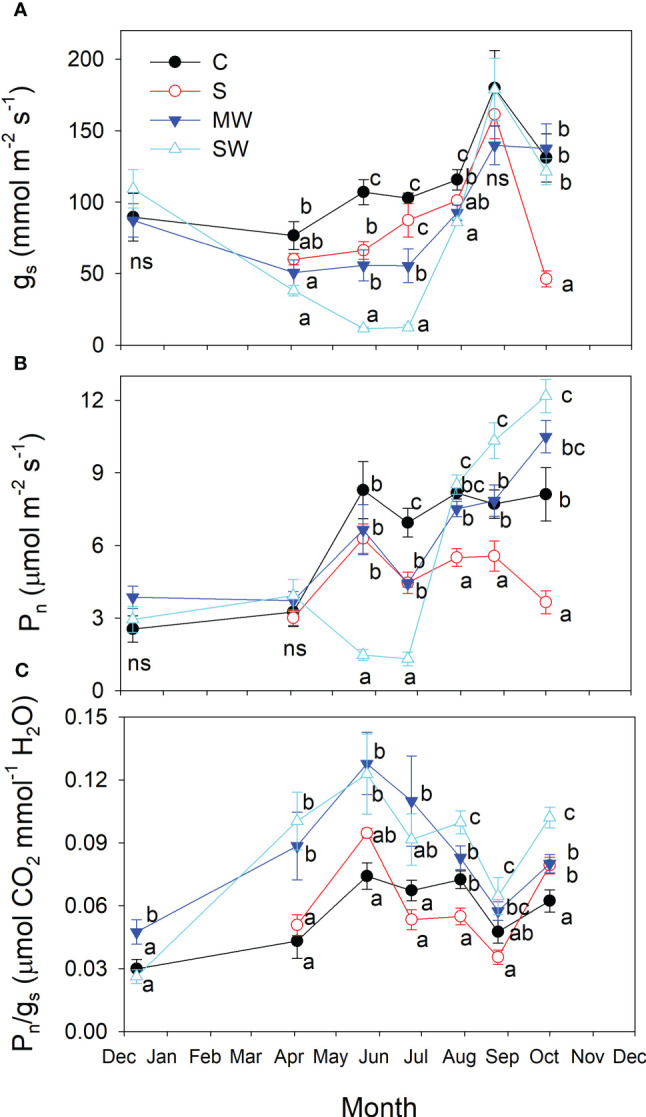
Evolution of stomatal conductance [g_s_; **(A)**], net photosynthetic rate [P_n_, **(B)**] and intrinsic water use efficiency [P_n_/g_s_; **(C)**] of *Pistacia lentiscus* plants subjected to saline and deficit irrigation treatments. Values are means of eight plants per treatment and vertical bars indicate SE. Symbols represent the different treatments: Control (filled circles), S (open circles), MW (filled triangles) and SW (open triangles). Different letters indicate significant differences between treatments according to Duncan t-test (P ≤ 0.05).

Salinity, and especially water deficit reduced stomatal conductance (g_s_) from the beginning of the experiment (**Figure 10A**), the effect being more pronounced in SW plants in the early summer (May and June). After which the values of g_s_ for water deficit plants recovered, reaching the control values. In contrast, differences in the g_s_ values with respect to the control produced by salinity were more marked at the end of the experiment, reaching g_s_ values very low at this time (**Figure 10A**). Such reductions in photosynthesis levels were also evident, although the differences observed were less pronounced (**Figure 10B**). The P_n_ values fell earlier and stronger in the severe water stress than in the saline treatment, but by the conclusion of the experiment, there was a notable decrease observed in this parameter disappeared in the water stressed plants, and MW and SW plants had higher P_n_ values than control plants. In general, the plants subjected to water stress treatments exhibited higher P_n_/g_s_ ratios, representing intrinsic water use efficiency, compared to the control plants throughout the duration of the experiment, as depicted in **Figure 10C**, but no pronounced differences were found between control and S, in the later treatment P_n_ and g_s_ were proportionally reduced.

## 4 Discussion

Climate change is affecting Mediterranean regions with extreme weather events, such as prolonged droughts and increases in soil salinity. These conditions can affect the capacity for grow and survive in plants, even for species that are adapted to these regions. Species that grow in Mediterranean regions, such as *Pistacia lentiscus* provide important ecosystem services such as ornamental production, soil protection, and carbon capture (Romano et al., 2022). Therefore, it is essential to find ways to protect and improve their capacity to adjust to changing conditions. The growth response of species to stresses is a complex expression of physiological and biochemical parameters. Although the tolerance mechanisms for salt and drought stress are similar physiologically and sometimes, they overlap, some aspects of a plant's physiology and metabolism may change if the plant is under salinity or water stress (Álvarez et al., 2018). In our conditions, we found that *Pistacia lentiscus* plants were more affected by deficit irrigation than by saline water. Concretely, different stresses induced different growth responses in *Pistacia lentiscus*, in this sense, both deficit irrigation treatments showed significant differences compared to the control and salinity in leaf/total DW and stem/total DW. However, the severe irrigation deficit treatments had differences in variables such as total DW, SLA (as measure of the proportion of plant biomass invested in leaf area but also of relative thickness, density or spread of leaves), LAR (as measure of photosynthetic surface relative to respiratory mass), or plant height (Wellstein et al., 2017; Gonzalez-Paleo and Ravetta, 2018).

Several variables and integrated them into a star and ray graph, which corroborated the differences between treatments indicated above were analysed. Aesthetically and commercially, customers appreciate a compact and architecturally balanced plant with increased foliage size in relation to plant height and with no alterations in leaf colour (Álvarez et al., 2013). Indeed, leaf colour is also an important attribute of ornamental plants, when used in gardening or landscaping projects, which influences its commercial value (Navarro et al., 2009). Customers associate yellow leaves with senescence, which is a negative trait that decrease the quality and attractiveness of the plant (Boutigny et al., 2020). In our conditions, no significant modifications in the leaf colour parameters were found and only treatment with moderate lack of irrigation had the highest value for the compactness variable. Therefore, the kind of stress should be taken into consideration as a key factor when using saline water and/or deficit irrigation as an irrigation strategy. This may affect the ornamental quality of the plants, assessed through growth parameters, to a greater or lesser extent (Koniarski and Matysiak, 2013; Sánchez-Blanco et al., 2019). Our results indicate that although both deficit irrigation and salinity reduced relative growth rate in *Pistacia lentiscus*, the time that each stress took to have an impact on plant growth differed significantly between salinity and water stress. Salinity affected growth much later than water stress, and such a reduction was only visible after a considerable amount of time had passed since the beginning of the irrigation treatments, as salts take time to accumulate inside plants before the concentrations reach dangerous levels and influence plant function (Munns and Tester, 2008). This underlines the fact that the duration of the water and salt stress is a crucial factor (Álvarez and Sánchez-Blanco, 2015; Vivaldi et al., 2021).

Sodium (Na^+^) and chloride (Cl^-^) are two ions that can accumulate in plant roots, particularly when plants are exposed to high levels of salts. When salt is present in the soil, the plant roots take up both water and salt. Sodium and chloride ions are particularly mobile and can easily move into the root cells. High concentrations of Na^+^ and Cl^-^ in the root cells can create an osmotic imbalance, leading to reduced water uptake and increased water stress. Additionally, high levels of Na^+^ in the plant can compete with other essential cations, such as potassium (K^+^) or calcium (Ca^2+^), for uptake and transport, leading to nutrient imbalances and potential toxicity (Acosta-Motos et al., 2016). A low K^+^/Na^+^ and Ca^2+^/Na^+^ ratios can cause several physiological changes in plants, such as reduced photosynthesis, nutrient imbalance, and oxidative stress, which can negatively affect plant growth and productivity. Sodium and potassium interact on two levels: interference with K^+^ nutrition and replacement of Na^+^ for K^+^ (Haro et al., 2010). High Na^+^ concentrations in plants cause K^+^ shortage symptoms and impair several K^+^-mediated physiological activities, including protein synthesis and enzymatic responses. Furthermore, membrane depolarization generated by Na^+^ input into the cell impairs K^+^ uptake through inward-rectifying K^+^ channels, making it thermodynamically unfavourable, as well as increased K^+^ outflow through outward-rectifying channels (Shabala et al., 2006; Coskun et al., 2013). Despite the controversy over whether NRT proteins transport NO^3–^, K^+^ or both, it is evident that Na^+^ competes with K^+^ in plant uptake specifically through High-Affinity K^+^/K^+^ uptake/K^+^ Transporter, High-Affinity Potassium Transporters and Non-Selective Cation Channel (Kronzucker and Britto, 2011). High-Affinity Potassium transporters are required for K^+^ uptake, root hair production and tolerance to abiotic stressors (Nieves-Cordones et al., 2014). They may, nevertheless, play a crucial function in facilitating Na^+^ absorption. Indeed, several members of this family have been demonstrated to mediate high-affinity Na+ uptake.

As a result of salt stress, some species have developed mechanisms to maintain a high K^+^/Na^+^ and Ca^2+^/Na^+^ ratios. These mechanisms include the selective uptake of K^+^ or Ca^2+^ over Na^+^, the exclusion of Na^+^ from uptake, the compartmentalization of Na^+^ in vacuoles in the root cells, and the transport of Na^+^ back to the soil through the salt glands or leaf excretion (Parihar et al., 2015; Isayenkov and Maathuis, 2019; Acosta-Motos et al., 2020). Maintaining a high K^+^/Na^+^ and Ca^2+^/Na^+^ ratios in plant tissues can be critical for plant survival under salinity stress. Therefore, the K^+^/Na^+^ and Ca^2+^/Na^+^ ratios can be used as a diagnostic tool to assess plant tolerance to salinity stress. By measuring the K^+^/Na^+^ and Ca^2+^/Na^+^ ratios in plant tissues, growers and researchers can gain insights into the plant's response to salinity stress and develop strategies to enhance plant tolerance to salinity stress (Acosta-Motos et al., 2014; Liu et al., 2019; Bello et al., 2021). In our study, the saline treatment (S) shows the highest values in Na^+^ and Cl^-^ ions and the lowest values for both ratios in leaves and stems. On the contrary, the severe deficit treatment (SW) showed the highest values in K^+^ and Ca^2+^ ions and both ratios also in the same organs. Also, this nutritional behaviour associated with the saline treatment (S) and severe water deficit treatment (SW) made it possible to separate them very well, especially because of the importance of K^+^ and Na^+^ and other nutrients as Mg^2+^ and Ca^2+^ in PC1 and due to the Na^+^ and Cl^-^ ions in PC2. Therefore, other nutrients in leaves, as Mg^2+^ or Ca^2+^, also are important. Mg ^2+^ can have a higher demand as a nutrient for chlorophyll synthesis (as it is part of its chemical structure), which is essential for carrying out a more efficient process of photosynthesis and increasing the water use efficiency (WUE) under stress situations (Dias et al., 2017; Tränkner et al., 2018; Ouled Youssef and Krouma, 2021). In our study, a higher concentration of Mg^2+^ in leaves could optimise the photosynthetic process despite increased stomata closure in the SW and MW treatments (especially in SW) contributing to an increase in WUE. Ca^2+^ is crucial to ensure the maintenance of cellular rigidity, as it plays a vital role in the stiffness and stability of cell walls (as it is part of their chemical composition). Ca^2+^ helps maintain the structural integrity of cells and prevents them from collapsing or being damaged under water stress conditions. Additionally, Ca^2+^can improve cellular turgor or maintain proper cellular turgor even under severe deficit irrigation conditions contributing to cell survival and normal functioning (Ahanger et al., 2013; Kour et al., 2023). In our study, a higher concentration of Ca^2+^ in leaves could assume the same roles as indicated above in the SW and MW treatments (especially in SW).

The relative water content (RWC) at predawn and midday are commonly used time points to assess changes in plant water status. RWC at predawn reflects plant water potential and indicates the level of water stress. Since photosynthesis and transpiration haven't started yet, there is no additional water loss during this time. On the other hand, RWC at midday can indicate plant water use efficiency. A higher RWC at midday suggests the plant maintains good hydration despite transpiration, while a lower RWC indicates water stress (Gindaba et al., 2005). In our study, moderate water deficit led to higher RWC values at predawn during summer months compared to the control. RWC values at midday were more affected by irrigation restriction, with lower values in severe treatments. Saline treatment showed little difference from the control. RWC is more sensitive to water stress than salt stress due to its definition as a measure of plant hydration. Water stress reduces RWC, while salinity stress hampers water absorption. RWC is a more rapid and sensitive indicator of water stress compared to plant growth and yield affected by salinity stress. RWC values in drought treatments recover after the hottest months, as cooler temperatures reduce water stress and enhance water uptake. Recovery depends on factors like severity and duration of stress, plant species, tolerance, and soil water availability (Toscano et al., 2019). During hot and dry months, water stress can cause a decline in RWC, potentially leading to plant damage or death (Alhaithloul, 2019). However, if the stress is not severe, the plant can recover RWC during cooler and wetter months (Galmés et al., 2007). Cooler temperatures reduce transpiration and slow water loss, maintaining or increasing RWC. They also increase soil water-holding capacity, supplying more water for plant uptake and aiding RWC recovery (Juenger and Verslues, 2023).

In a similar way to RWC, leaf water potential can vary throughout the day due to changes in plant water status, with different values typically observed at predawn and midday and it has also demonstrated usefulness as a stress index in a large number of species (Álvarez et al., 2019; Sánchez-Blanco et al., 2019; Álvarez et al., 2020). The difference between the leaf water potential at predawn and midday can be used to evaluate the plant's ability to maintain its water status throughout the day. A smaller difference between the two values indicates that the plant is able to maintain its water status during the day and that it has good water use efficiency, while a larger difference indicates that the plant is experiencing water stress during the day and that it may not be using water efficiently. In our study, as one would expect the changes in leaf water potential were more evident in predawn that in midday due to the influence of environmental factors as mentioned above and more pronounced in drought treatments during the summer months.

During drought and saline conditions, the availability of water in the soil decreases, and plants often experience water stress and as a result, the stem water potential of the plant also decreases (Pérez-Pérez et al., 2009; Yang et al., 2020). When water is readily available in the soil, the plant takes up water through its roots and this water is transported through the plant's vascular system to the leaves. This movement of water is driven by differences in water potential between the soil, the plant, and the atmosphere (Berry et al., 2019; Scharwies and Dinneny, 2019). When soil water availability decreases due to drought or salinity, the plant's ability to take up water is reduced, and water transport through the plant is also affected. As the plant attempts to cope with water or salt stress, it may reduce its transpiration rate and water use to conserve water (Bertolino et al., 2019). This could result in a decline in stem water potential as the plant attempts to maintain water balance and avoid tissue damage (Levy et al., 2013; Lugassi et al., 2019). In some cases, the stem water potential may decrease to a level that is so low that the plant wilts or even dies (Abideen et al., 2021). In our study, depending on the month analysed and especially in the summer months, sometimes it is the saline treatment or the severe deficit irrigation, the treatments that show the lowest values in stem water potential. To cope with low stem water potential, plants can develop various mechanisms to optimize water use and minimize water loss. Some of these mechanisms include stomatal regulation, root and leaf adaptations, dormancy and shedding, or cellular protection. In our study, *Pistacia lentiscus* plants regulated the opening and closing of stomata to control water loss through transpiration and plants subjected to saline and water deficit produced osmoprotectants and antioxidants in order to help protect cells from damage caused by dehydration and oxidative stress. These mechanisms collectively help plants survive and adapt to low stem water potential by reducing water loss, optimizing water uptake, and maintaining essential physiological processes as much as possible during challenging conditions.

Plants increase their osmotic adjustment in response to salt or drought stress, which results in the accumulation of osmolytes like soluble sugars, amino acids, and organic acids, in plant cells in response to a depletion of the available soil moisture (Callister et al., 2006; Singh et al., 2015; Khan et al., 2020). This allows the plant to maintain its turgor pressure and prevent wilting. Osmotic adjustment is closely related to the leaf water potential and relative water content of the plant. Maintaining high RWC at low leaf water potentials is primarily related to osmotic adjustment, which could be considered as a strategy to tolerate water stress (Pérez-Pérez et al., 2009; Banks and Hirons, 2019). In our study, *P. lentiscus* plants submitted to water stress and salinity showed osmotic adjustment, which was more pronounced under salinity than under both levels of water deficit. NaCl-induced osmotic adjustment is also a common response to salinity stress caused by high concentrations of salt in the soil or irrigation water (Zhang et al., 2022). To counteract the decrease in water availability due to high salt concentration, plants can adjust their osmotic potential by accumulating solutes, sugars, and amino acids, in their cells. However, the effectiveness of osmotic adjustment in alleviating the effects of salinity stress depends on the plant species and cultivars, as well as the concentration and duration of the salt stress (Liao et al., 2022; Egea et al., 2023). In our study, *Pistacia lentiscus* might exhibit some tolerance to a salinity level of 4 dS m^-1^, but it's essential to remember that "tolerance" doesn't mean thriving. Even tolerant plants can show signs of stress and reduced growth under elevated salt conditions. *Pistacia lentiscus* is known to have some degree of salt tolerance, but its exact response to different salinity levels can vary. In our study, *P lentiscus* plants irrigated with salinity level of 4 dS m^-1^ during 11 months reduced growth, altered nutrient uptake, increased the osmotic adjustment and reduced gas exchange parameters.

The findings from our study indicate that the observed reduction in g_s_ is an adaptive and efficient mechanism used by the species to control transpiration (Hatfield and Dold, 2019). This mechanism helps the plant can withstand salinity and water stress, particularly during periods of high transpiration. In the case of plants under deficit irrigation conditions, this mechanism limits water loss (Lefi et al., 2023). For saline plants, the reduction in g_s_ helps to decrease the salt load on leaves, thus increasing longevity by keeping salts at subtoxic levels for a longer time (Izadi et al., 2022). The delay in the reduction of g_s_ in saline plants as compared to deficit irrigation plants is due to the time it takes for salts to accumulate inside the plant before reaching toxic levels that could affect plant functioning (Munns, 2011; Pirasteh-Anosheh et al., 2016). Similar changes in P_n_ were observed also in g_s_ between the treatments studied. The *P. lentiscus* plants that were subjected to both deficit irrigation treatments exhibited an increase in their intrinsic water use efficiency (P_n_/g_s_), particularly during periods of high-water demand. An increase in intrinsic water use efficiency (P_n_/g_s_) in plants indicates that they are able to photosynthesize and produce the same amount of biomass while using less water, which is an important adaptation for plants growing in environments with water scarcity and salt stress (Fernández-García et al., 2014; Liao et al., 2022). Plants face a constant dilemma: to die of thirst or to die of hunger. This is because plants need water to carry out photosynthesis and produce energy, but they also need nutrients such as nitrogen, phosphorus, and potassium to grow and develop. If plants do not receive enough water, their ability to photosynthesize is compromised, reducing their energy production capacity and ultimately leading to plant death by dehydration (Chaves et al., 2016). On the other hand, if plants do not receive enough nutrients, their growth is affected, and their energy production capacity is also reduced, leading to death by starvation. To survive, plants must find a balance between water and nutrient uptake and water loss through transpiration. This is achieved by regulating stomatal aperture, which controls the entry and exit of water and gases in the plant (Bhattacharya, 2021). While this balance may vary depending on environmental conditions and plant species, most plants have evolved to optimize their efficiency in using water and nutrients and to adapt to variable conditions in their environment.

It can be inferred that the application of moderate and severe deficit irrigation (60 and 40 % reductions with respect to the control) and the use of saline water with a determined level of salinity (around 4 dS m^-1^) is feasible for growing this species. Based on its observed behaviour when applying deficit irrigation strategies and irrigation with low quality water, *Pistacia lentiscus* is proposed as a suitable species for gardening projects and landscaping in arid and saline areas

## Data availability statement

The original contributions presented in the study are included in the article/**Supplementary Material**, further inquiries can be directed to the corresponding author.

## Author contributions

SA performed the experiment and drafted the manuscript. MS-B and SA designed and instructed the research work. SA and JA-M carried out statistical analysis. MS-B also coordinated the study and provided study material and facilities for the experiments. The three authors were involved in data interpretation, paper preparing and article writing. All authors have read and approved the final manuscript.

## References

[B1] Abideen Z. Koyro H. W. Huchzermeyer B. Ahmed M. Zulfiqar F. Egan T.. (2021). *Phragmites karka* plants adopt different strategies to regulate photosynthesis and ion flux in saline and water deficit conditions. Plant Biosystems-An Int. J. Dealing all Aspects Plant Biol. 155, 524–534. doi: 10.1080/11263504.2020.1762783

[B2] Acosta-Motos J. R. Álvarez S. Hernández J. A. Sánchez-Blanco M. J. (2014). Irrigation of *Myrtus communis* plants with reclaimed water: Morphological and physiological responses to different levels of salinity. J. Hortic. Sci. Biotechnol. 89, 487–494. doi: 10.1080/14620316.2014.11513110

[B3] Acosta-Motos J. R. Ortuño M. F. Álvarez S. López-Climent M. F. Gómez-Cadenas A. Sánchez-Blanco M. J. (2016). Changes in growth, physiological parameters and the hormonal status of *Myrtus communis* L. plants irrigated with water with different chemical compositions. J. Plant Physiol. 191, 12–21. doi: 10.3390/agronomy7010018 26703779

[B4] Acosta-Motos J. R. Ortuño M.F. Bernal-Vicente A. Díaz-Vivancos P. Sánchez-Blanco M. J. Hernández J. A.. (2017). Plant responses to salt stress: Adaptive mechanisms. Agronomy 7, 18. doi: 10.3390/agronomy7010018

[B5] Acosta-Motos J. R. Penella C. Hernández J. A. Díaz-Vivancos P. Sánchez-Blanco M. J. Navarro J. M.. (2020). Towards a sustainable agriculture: Strategies involving phytoprotectants against salt stress. Agronomy 10, 194. doi: 10.3390/agronomy10020194

[B6] Ahanger M. A. Tyagi S. R. Wani M. R. Ahmad P. (2013). “Drought tolerance: role of organic osmolytes, growth regulators, and mineral nutrients,” in Physiological mechanisms and adaptation strategies in plants under changing environment, vol. 1. (New York, NY: Springer New York), 25–55).

[B7] Albatayneh A. (2023). Water energy food nexus to tackle climate change in the eastern mediterranean. Air Soil Water Res. 16, 11786221231170222. doi: 10.1177/11786221231170222

[B8] Alhaithloul H. A. S. (2019). Impact of combined heat and drought stress on the potential growth responses of the desert grass *Artemisia sieberi* alba: Relation to biochemical and molecular adaptation. Plants 8 (10), 416. doi: 10.3390/plants8100416 31618849 PMC6843163

[B9] Álvarez S. Bañón S. Sánchez-Blanco M. J. (2013). Regulated deficit irrigation indifferent phonological stages of potted geranium plants: water consumption, water relations and ornamental quality. Acta Physiol. Plant 35, 1257–1267. doi: 10.1007/s11738-012-1165-x

[B10] Álvarez S. Gómez-Bellot M. J. Acosta-Motos J. R. Sánchez-Blanco M. J. (2019). Application of deficit irrigation in *Phillyrea angustifolia* for landscaping purposes. Agric. Water Manage. 218, 193–202. doi: 10.1016/j.agwat.2019.03.049

[B11] Álvarez S. Martín H. Barajas E. Rubio J. A. Vivaldi G. A. (2020). Rootstock effects on water relations of young almond trees (cv. Soleta) when subjected to water stress and rehydration. Water 12, 3319. doi: 10.3390/w12123319

[B12] Álvarez S. Rodriguez P. Broetto F. Sánchez-Blanco M. J. (2018). Long term responses and adaptive strategies of *Pistacia lentiscus* under moderate and severe deficit irrigation and salinity: Osmotic and elastic adjustment, growth, ion uptake and photosynthetic activity. Agric. Water Manage. 202, 253–262. doi: 10.1016/j.agwat.2018.01.006

[B13] Álvarez S. Sánchez-Blanco M. J. (2015). Comparison of individual and combined effects of salinity and deficit irrigation on physiological, nutritional and ornamental aspects of tolerance in *Callistemon laevis* plants. J. Plant Physiol. 185, 65–74. doi: 10.1016/j.jplph.2015.07.009 26277754

[B14] Arve L. E. Torre S. Olsen J. E. Tanino K. K. (2011). “Stomatal responses to drought stress and air humidity,” in Abiotic stress in plants-Mechanisms and adaptations. Shanker A, editor. (IntechOpen). Available at: 10.5772/895

[B15] Asif Z. Chen Z. Sadiq R. Zhu Y. (2023). Climate change impacts on water resources and sustainable water management strategies in north america. Water Resour. Manage. 37, 2771–2786. doi: 10.1007/s11269-023-03474-4

[B16] Banks J. M. Hirons A. D. (2019). Alternative methods of estimating the water potential at turgor loss point in *Acer* genotypes. Plant Methods 15, 34. doi: 10.1186/s13007-019-0410-3 30988693 PMC6448274

[B17] Barrs H. D. (1968). “Determination of water deficits in plants tissue,” in Water deficits and plant growth. Ed. Kozlowski T. T. (New York, NY, USA: Academic Press), 235–368.

[B18] Begg J. E. Turner N. C. (1970). Water potential gradients in field tobacco. Plant Physiol. 46, 343–346. doi: 10.1104/pp.46.2.343 16657462 PMC396591

[B19] Bello S. K. Alayafi A. H. AL-Solaimani S. G. Abo-Elyousr K. A. (2021). Mitigating soil salinity stress with gypsum and bio-organic amendments: A review. Agronomy 11, 1735. doi: 10.3390/agronomy11091735

[B20] Berry Z. C. Emery N. C. Gotsch S. G. Goldsmith G. R. (2019). Foliar water uptake: processes, pathways, and integration into plant water budgets. Plant Cell Environ. 42, 410–423. doi: 10.1111/pce.13439 30194766

[B21] Bertolino L. T. Caine R. S. Gray J. E. (2019). Impact of stomatal density and morphology on water-Use efficiency in a changing world. Front. Plant Sci. 10. doi: 10.3389/fpls.2019.00225 PMC6414756 30894867

[B22] Bhattacharya A. (2021). Effect of soil water deficits on plant–water relationship: A review. In Soil Water Deficit and Physiological Issuesin Plants; Springer: Singapore; pp. 1–98. doi: 10.1007/978-981-33-6276-5_1

[B23] Bodner G. Nakhforoosh A. Kaul H. P. (2015). Management of crop water under drought: a review. Agron. Sustain. Dev. 35, 401–442. doi: 10.1007/s13593-015-0283-4

[B24] Boutigny A. L. Dohin N. Pornin D. Rolland M. (2020). Overview and detectability of the genetic modifications in ornamental plants. Horticulture Res. 7, 11. doi: 10.1038/s41438-019-0232-5 PMC6994484 32025314

[B25] Bussotti F. Ferrini F. Pollastrini M. Fini A. (2014). The challenge of Mediterranean sclerophyllous vegetation under climate change: From acclimation to adaptation. Environ. Exp. Bot. 103, 80–98. doi: 10.1016/j.envexpbot.2013.09.013

[B26] Callister A. N. Arndt S. K. Adams M. A. (2006). Comparison of four methods for measuring osmotic potential of tree leaves. Physiol. Plant 127, 383–392. doi: 10.1111/j.1399-3054.2006.00652.x

[B27] Castillo-Campohermoso M. A. Broetto F. Rodríguez-Hernández A. M. Soriano-Melgar L. D. A. A. Mounzer O. Sánchez-Blanco M. J. (2020). Plant-available water, stem diameter variations, chlorophyll fluorescence, and ion content in *Pistacia lentiscus* under salinity stress. Terra Latinoamericana 38 (1), 103–111. doi: 10.28940/terra.v38i1.510

[B28] Chaudhry S. Sidhu G. P. S. (2022). Climate change regulated abiotic stress mechanisms in plants: A comprehensive review. Plant Cell Rep. 41, 1–31. doi: 10.1007/s00299-021-02759-5 34351488

[B29] Chaves M. M. Costa J. M. Zarrouk O. Pinheiro C. Lopes C. M. Pereira J. S. (2016). Controlling stomatal aperture in semi-arid regions—The dilemma of saving water or being cool? Plant Sci. 251, 54–64. doi: 10.1016/j.plantsci.2016.06.015 27593463

[B30] Coskun D. Britto D. T. Jean Y. K. Kabir I. Tolay I. Torun A. A.. (2013). K(+) efflux and retention in response to NaCl stress do not predict salt tolerance in contrasting genotypes of rice (Oryza sativa L.). PloS One 8, e57767. doi: 10.1371/journal.pone.0057767 23460903 PMC3583904

[B31] Dias K. G. D. L. Guimarães P. T. G. Furtini Neto A. E. Silveira H. R. O. D. Lacerda J. J. D. J (2017). Effect of magnesium on gas exchange and photosynthetic efficiency of coffee plants grown under different light levels. Agriculture 7, 85. doi: 10.3390/agriculture7100085

[B32] Egea I. Estrada Y. Faura C. Egea-Fernández J. M. Bolarin M. C. Flores F. B. (2023). Salt-tolerant alternative crops as sources of quality food to mitigate the negative impact of salinity on agricultural production. Front. Plant Sci. 14. doi: 10.3389/fpls.2023.1092885 PMC9935836 36818835

[B33] Fernández-García N. Olmos E. Bardisi E. García-De la Garma J. López-Berenguer C. Rubio-Asensio J. S. (2014). Intrinsic water use efficiency controls the adaptation to high salinity in a semi-arid adapted plant, henna (*Lawsonia inermis* L.). J. Plant Physiol. 171, 64–75. doi: 10.1016/j.jplph.2013.11.004 24484959

[B34] Galmés J. Flexas J. Savé R. Medrano H. (2007). Water relations and stomatal characteristics of Mediterranean plants with different growth forms and leaf habits: responses to water stress and recovery. Plant Soil 290, 139–155. doi: 10.1007/s11104-006-9148-6

[B35] Gindaba J. Rozanov A. Negash L. (2005). Photosynthetic gas exchange, growth and biomass allocation of two *Eucalyptus* and three indigenous tree species of Ethiopia under moisture deficit. For. Ecol. Manage. 205, 127–138. doi: 10.1016/j.foreco.2004.10.056

[B36] Gonzalez-Paleo L. Ravetta D. A. (2018). Relationship between photosynthetic rate, water use and leaf structure in desert annual and perennial forbs differing in their growth. Photosynthetica 56, 1177–1187. doi: 10.1007/s11099-018-0810-z

[B37] Gucci R. Xyloyannis C. Flore J. A. (1991). Gas exchange parameters, water relations and carbohydrates partitioning in leaves of field-grown *Prunus domestica* following fruit removal. Physiol. Plant 83, 497–505. doi: 10.1111/j.1399-3054.1991.tb00126.x

[B38] Haro R. Banuelos M. A. Rodriguez-Navarro A. (2010). High-affinity sodium uptake in land plants. Plant Cell Physiol. 51, 68–79. doi: 10.1093/pcp/pcp168 19939835

[B39] Hatfield J. L. Dold C. (2019). Water-use efficiency: advances and challenges in a changing climate. Front. Plant Sci. 10. doi: 10.3389/fpls.2019.00103 PMC6390371 30838006

[B40] Hotelling H. (1933). Analysis of a complex of statistical variables into principal components. J. Educ. Psychol. 24, 417–441. doi: 10.1037/h0071325

[B41] Isayenkov S. V. Maathuis F. J. M. (2019). Plant salinity stress: many unanswered questions remain. Front. Plant Sci. 10. doi: 10.3389/fpls.2019.00080 PMC6384275 30828339

[B42] Izadi Y. Moosavi S. A. Gharineh M. H. (2022). Salinity affects eco-physiological aspects and biochemical composition in chia (*Salvia hispanica* L.) during germination and seedling growth. Sci. Hortic. 306, 111461. doi: 10.1016/j.scientia.2022.111461

[B43] Juenger T. E. Verslues P. E. (2023). Time for a drought experiment: Do you know your plants’ water status? Plant Cell 35, 10–23. doi: 10.1093/plcell/koac324 36346190 PMC9806650

[B44] Khan N. Ali S. Zandi P. Mehmood A. Ullah S. Ikram M.. (2020). Role of sugars, amino acids and organic acids in improving plant abiotic stress tolerance. Pak. J. Bot. 52, 355–363. doi: 10.30848/PJB2020-2(24

[B45] Koniarski M. Matysiak B. (2013). Growth and development of potted rhododendron cultivars” Catawbiense Boursault” and “Old Port” in response to regulated deficit irrigation. J. Hortic. Res. 21 , 29–37. doi: 10.2478/johr-2013-0005

[B46] Kour J. Khanna K. Singh A. D. Dhiman S. Bhardwaj T. Devi K.. (2023). Calcium’s multifaceted functions: From nutrient to secondary messenger during stress. South Afr. J. Bot. 152, 247–263. doi: 10.1016/j.sajb.2022.11.048

[B47] Kronzucker H. J. Britto D. T. (2011). Sodium transport in plants: a critical review. New Phytol. 189, 54–81. doi: 10.1111/j.1469-8137.2010.03540.x 21118256

[B48] Lefi E. Zorrig W. Hamed S. B. Rabhi M. Abdelly C. Chaieb M. (2023). Photosynthetic behaviour of *Hedysarum carnosum* and *Hedysarum coronarium* under drought stress. Acta Physiol. Plant 45, 79. doi: 10.1007/s11738-023-03560-5

[B49] Leotta L. Toscano S. Ferrante A. ROmano D. Francini A. (2023). New strategies to increase the abiotic stress tolerance in woody ornamental plants in mediterranean climate. Plants 12, 2022. doi: 10.3390/plants12102022 37653939 PMC10223706

[B50] Levy D. Coleman W. K. Veilleux R. E. (2013). Adaptation of potato to water shortage: irrigation management and enhancement of tolerance to drought and salinity. Am. J. Potato Res. 90, 186–206. doi: 10.1007/s12230-012-9291-y

[B51] Liao Q. Gu S. Kang S. Du T. Tong L. Wood J. D.. (2022). Mild water and salt stress improve water use efficiency by decreasing stomatal conductance via osmotic adjustment in field maize. Sci. Total Environ. 805, 150364. doi: 10.1016/j.scitotenv.2021.150364 34818800

[B52] Liu C. Zhao X. Yan J. Yuan Z. Gu M. (2019). Effects of salt stress on growth, photosynthesis, and mineral nutrients of 18 pomegranate (*Punica granatum*) cultivars. Agronomy 10, 27. doi: 10.3390/agronomy10010027

[B53] Lugassi N. Yadav B. S. Egbaria A. Wolf D. Kelly G. Neuhaus E.. (2019). Expression of *Arabidopsis hexokinase* in tobacco guard cells increases water-use efficiency and confers tolerance to drought and salt stress. Plants 8, 613. doi: 10.3390/plants8120613 31888275 PMC6963886

[B54] McGuire R. G. (1992). Reporting of objective colour measurements. HortScience 27, 1254–1255. doi: 10.21273/HORTSCI.27.12.1254

[B55] Munns R. (2011). Plant adaptations to salt and water stress: differences and commonalities. Adv. Botanical Res. 57, 1–32. doi: 10.1016/B978-0-12-387692-8.00001-1

[B56] Munns R. Tester M. (2008). Mechanisms of salinity tolerance. Annu. Rev. Plant Biol. 59, 651–681. doi: 10.1146/annurev.arplant.59.032607.092911 18444910

[B57] Navarro A. Álvarez S. Castillo M. Bañón S. Sánchez-Blanco M. J. (2009). Changes in tissue-water relations, photosynthetic activity, and growth of *Myrtus communis* plants in response to different conditions of water availability. J. Hortic. Sci. Biotech. 84, 541–5547. doi: 10.1080/14620316.2009.11512563

[B58] Nieves-Cordones M. Aleman F. Martinez V. Rubio F. (2014). K(+) uptake in *plant roots. The systems involved, their regulation and parallels in other organisms* . J. Plant Physiol. 171, 688–695. doi: 10.1016/j.jplph.2013.09.021 24810767

[B59] Ouled Youssef I. Krouma A. (2021). Functional dissection of magnesium nutrition and use efficiency in common bean. Agron. J. 113, 261–269. doi: 10.1002/agj2.20506

[B60] Paranychianakis N. V. Chartzoulakis K. S. (2005). Irrigation of Mediterranean crops with saline water: from physiology to management practices. Agriculture Ecosyst. Environ. 106, 171–187. doi: 10.1016/j.agee.2004.10.006

[B61] Parihar P. Singh S. Singh R. Singh V. P. Prasad S. M. (2015). Effect of salinity stress on plants and its tolerance strategies: a review. Environ. Sci. pollut. Res. Int. 22, 4056–4075. doi: 10.1007/s11356-014-3739-1 25398215

[B62] Parraga-Aguado I. Gonzalez-Alcaraz M. N. Alvarez-Rogel J. Jimenez-Carceles F. J. Conesa H. M. (2013). The importance of edaphic niches and pioneer plant species succession for the phytOmanagement of mine tailings. Environ. pollut. 176, 134–143. doi: 10.1016/j.envpol.2013.01.023 23419771

[B63] Patel R. Mukherjee S. Gosh S. Sahu B. (2023). “Climate risk management in dryland agriculture: technological management and institutional options to adaptation,” in Enhancing resilience of dryland agriculture under changing climate: interdisciplinary and convergence approaches (Singapore: Springer Nature Singapore), 55–73.

[B64] Pearson K. (1901). On lines and planes of closet fit to systems of points in space. Philos. Magazine 2, 559–572. doi: 10.1080/14786440109462720

[B65] Pérez-Pérez J. G. Robles J. M. Tovar J. C. Botía P. (2009). Response to drought and salt stress of lemon ‘Fino 49’under field conditions: water relations, osmotic adjustment and gas exchange. Sci. Hortic. 122, 83–90. doi: 10.1016/j.scienta.2009.04.009

[B66] Phogat V. Mallants D. Cox J. W. Šimůnek J. Oliver D. P. Awad J. (2020). Management of soil salinity associated with irrigation of protected crops. Agric. Water Manage. 227, 105845. doi: 10.1016/j.agwat.2019.105845

[B67] Pirasteh-Anosheh H. Ranjbar G. Pakniyat H. Emam Y. (2016). Physiological mechanisms of salt stress tolerance in plants: An overview. Plant-Environment Interaction: Responses Approaches to Mitigate Stress, 141–160. doi: 10.1002/9781119081005.ch8

[B68] Ramón Vallejo V. Smanis A. Chirino E. Fuentes D. Valdecantos A. Vilagrosa A. (2012). Perspectives in dryland restoration: approaches for climate change adaptation. New Forests 43, 561–579. doi: 10.1007/s11056-012-9325-9

[B69] Romano G. Ricci G. F. Leronni V. Venerito P. Gentile F. (2022). Soil bioengineering techniques for Mediterranean coastal dune restoration using autochthonous vegetation species. J. Coast. Conserv. 26, 71. doi: 10.1007/s11852-022-00912-0

[B70] Sánchez-Blanco M. J. Ortuño M. F. Bañón S. Álvarez S. (2019). Deficit irrigation as a strategy to control growth in ornamental plants and enhance their ability to adapt to drought conditions. J. Hortic. Sci. Biotech. 94, 137–150. doi: 10.1080/14620316.2019.1570353

[B71] Scharwies J. D. Dinneny J. R. (2019). Water transport, perception, and response in plants. J. Plant Res. 132, 311–324. doi: 10.1007/s10265-019-01089-8 30747327

[B72] Scholander P. F. Hammel H. T. Bradstreet E. D. Hemingsen E. A. (1965). Sap pressure in vascular plants. Science 148, 339–346. doi: 10.1126/science.148.3668.339 17832103

[B73] Semeraro T. Scarano A. Leggieri A. Calisi A. De Caroli M. (2023). Impact of climate change on agroecosystems and potential adaptation strategies. Land 12, 1117. doi: 10.3390/land12061117

[B74] Shabala S. Demidchik V. Shabala L. Cuin T. A. Smith S. J. Miller A. J.. (2006). Extracellular Ca(2+) ameliorates NaCl-induced K(+) loss from Arabidopsis root and leaf cells by controlling plasma membrane K(+) -permeable channels. Plant Physiol. 141, 1653–1665. doi: 10.1104/pp.106.082388 16798942 PMC1533937

[B75] Singh M. Kumar J. Singh S. Singh V. P. Prasad S. M. (2015). Roles of osmoprotectants in improving salinity and drought tolerance in plants: a review. Rev. Environ. Sci. Biotechnol. 14, 407–426. doi: 10.1007/s11157-015-9372-8

[B76] Soares D. Paço T. A. Rolim J. (2022). Assessing climate change impacts on irrigation water requirements under mediterranean conditions—A review of the methodological approaches focusing on maize crop. Agronomy 13, 117. doi: 10.3390/agronomy13010117

[B77] Toscano S. Ferrante A. ROmano D. (2019). Response of Mediterranean ornamental plants to drought stress. Horticulturae 5 , 6. doi: 10.3390/horticulturae5010006

[B78] Tränkner M. Tavakol E. Jákli B. (2018). Functioning of potassium and magnesium in photosynthesis, photosynthate translocation and photoprotection. Physiol. Plant 163, 414–431. doi: 10.1111/ppl.12747 29667201

[B79] Turner N. C. (1988). Measurement of plant water status by the pressure chamber technique. Irri. Sci. 9, 289–308. doi: 10.1007/BF00296704

[B80] Vivaldi G. A. Camposeo S. Romero-Trigueros C. Pedrero F. Caponio G. Lopriore G.. (2021). Physiological responses of almond trees under regulated deficit irrigation using saline and desalinated reclaimed water. Agric. Water Manage. 258, 107172. doi: 10.1016/j.agwat.2021.107172

[B81] Wellstein C. Poschlod P. Gohlke A. Chelli S. Campetella G. Rosbakh S.. (2017). Effects of extreme drought on specific leaf area of grassland species: A meta-analysis of experimental studies in temperate and sub-Mediterranean systems. Glob. Change Biol. 23, 2473–2481. doi: 10.1111/gcb.13662 28208238

[B82] Williams J. T. (2002). Global research on underutilized crops: An assessment of current activities and proposals for enhanced cooperation (Bioversity International), 46. Available at: https://hdl.handle.net/10568/105302.

[B83] Yang Z. Li J.-L. Liu L.-N. Xie Q. Sui N. (2020). Photosynthetic regulation under salt stress and salt-tolerance mechanism of sweet sorghum. Front. Plant Sci. 10. doi: 10.3389/fpls.2019.01722 PMC6974683 32010174

[B84] Zhang X. Yang H. Du T. (2022). Osmotic adjustment of tomato under mild soil salinity can enhance drought resistance. Environm. Exp. Bot. 202, 105004. doi: 10.1016/j.envexpbot.2022.105004

