# Supplementary material for: Morphological performance and seasonal pattern of water relations and gas exchange in Pistacia lentiscus plants subjected to salinity and water deficit
Source: Front Plant Sci. 2023 Sep 5;14:1237332. doi: 10.3389/fpls.2023.1237332 (PMC10508188; doi:10.3389/fpls.2023.1237332)
Supplement: Supplementary file 1 [file DataSheet_1.pdf]

Supplementary table 1. Principal Component Analysis

Component Number	*Eugenvalue*	*Percentage of Variance*	*Percentage Cumulative*
1	5.68907	56.891	56.891
2	1.41824	14.182	71.073
3	0.888973	8.890	79.963
4	0.65639	6.564	86.527
5	0.535975	5.360	91.886
6	0.320805	3.208	95.095
7	0.209242	2.092	97.187
8	0.129417	1.294	98.481
9	0.0909508	0.910	99.391
10	0.0609369	0.609	100.000

Supplementary figure 1. Sedimentation graph

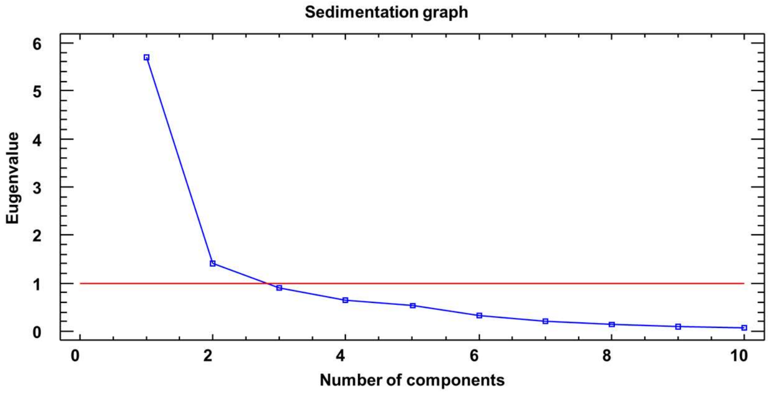

Supplementary table 2a. This table shows the scores of the principal components

Row	*Label*	*Component 1*	*Component 2*	*Average Component 1*	*Average Component 2*
1	C	-0.581192	-1.31394	**0.52636689**	**-1.1361861**
2	C	0.400885	-1.02475		
3	C	0.148435	-0.936265		
4	C	-0.147315	-1.541		
5	C	1.11923	-0.801087		
6	C	0.663806	-1.56608		
7	C	0.49889	-1.55068		
8	C	2.38381	-0.182868		
9	C	0.0771949	-0.865871		
10	C	0.699925	-1.57932		
11	S	2.989	0.364174	**3.087523**	**1.0292095**
12	S	2.43028	1.32726		
13	S	3.53892	1.18901		
14	S	2.8679	1.44399		
15	S	3.42511	1.27241		
16	S	2.80395	-0.832963		
17	S	3.39106	1.74125		
18	S	2.53895	1.68432		
19	S	3.93572	2.3143		
20	S	2.95434	-0.211656		
21	MW	-0.686808	-1.38944	**-0.53627734**	**-0.62336111**
22	MW	-0.817163	0.31904		
23	MW	-0.448246	0.12478		
24	MW	0.0242626	-0.354185		
25	MW	-1.1911	-0.0524861		
26	MW	1.18312	-1.90102		
27	MW	-1.852	-0.149207		
28	MW	-0.657048	-0.212678		
29	MW	0.189339	-1.8579		
30	MW	-1.10713	-0.760515		
31	SW	-2.43164	0.288159	**-3.077612**	**0.73033825**
32	SW	-4.02236	1.54556		
33	SW	-1.58196	-0.0290384		
34	SW	-2.73414	0.968858		
35	SW	-4.77477	2.6315		
36	SW	-3.98557	0.230813		
37	SW	-3.55475	0.217427		
38	SW	-3.06183	0.654938		
39	SW	-1.06897	-0.0831831		
40	SW	-3.56013	0.878349		

Supplementary table (ST) 2b. ANOVA table for the component 1 scores according to the treatments

Source	*Sum of squares*	*FD*	*Mean Square*	*F-Ratio*	*P-Value*
Intergroup	195.691	3	65.2305	89.69	0.0000
Intragroup	26.1821	36	0.727281		
Total (Corr.)	221.874	39			

Supplementary table (ST) 2c. Multiple comparisons test for the component 1 scores by treatments using Duncan method

Treatments	*Replicates*	*Mean*	*Homogenous groups*
se	10	-3.07761	d
m	10	-0.536277	c
c	10	0.526367	b
s	10	3.08752	a

Supplementary table (ST) 2d. ANOVA table for the component 2 scores according to the treatments

Source	*Sum of squares*	*FD*	*Mean Square*	*F-Ratio*	*P-Value*
Intergroup	32.7216	3	10.9072	17.38	0.0000
Intragroup	22.5899	36	0.627496		

Supplementary table (ST) 2e. Multiple comparisons test for the component 2 scores by treatments using Duncan method

Treatments	*Replicates*	*Mean*	*Homogenous groups*
c	10	-1.13619	b
m	10	-0.623361	b
se	10	0.730338	a
s	10	1.02921	a

Supplementary table 3. Table of Component Weights

Variables	*Component 1*	*Component 2*
Na	0.306514	**0.49968**
Cl	0.266487	**0.518413**
Ca	**-0.360976**	0.119107
K	-0.356106	-0.02951
B	-0.180505	**-0.413806**
Mg	**-0.371618**	0.0493698
Mn	-0.325627	**0.41141**
P	-0.333607	0.280477
S	-0.357117	0.126466
Zn	-0.249755	0.170054
